# Do Medical Colleges Strive to Thoroughly Educate Their Students?

**Published:** 1899-06

**Authors:** G. Lenox Curtis

**Affiliations:** New York.


ARTICLE V.

### DO MEDICAL COLLEGES STRIVE TO THOROUGHLY EDUCATE THEIR STUDENTS?

G. LENOX CURTIS, M. D., NEW YORK.

In considering this subject, especially during the past year, I have reached the conclusion that these institutions are conducted largely for personal gain and aggrandisement, and any innovation which might tend to defeat their purpose is frowned upon to their discredit, and to the detriment of their pupils. If knowledge is the acme of education why do not the presidents of colleges see that men best fitted for certain positions are placed in them? Is it because their own prominence depends on those members of the faculty who oppose investigation and research of new methods set forth by an unassuming practitioner, who has spent his hard earned dollars and years of his life in original investigation? A new theory, backed by scientific experiments, is rarely favorably received, unless advanced by men at the head of the profession—to follow whom is the fad of the day.

The medical profession is full of young men who have for years been working on original subjects with astontshing results, but fear of failure of recognition and unjust criticism have prevented them from making public their discoveries. If American colleges would cast politics aside, and open their doors to such investigations, this country would lead the world in medical science. Could this condition exist, the tide of students flowing to European colleges would be turned, and foreigners would finish their education in America.

What Koch so ignominiously failed in, the treatment of tuberculosis has been accomplished in America, but the profession frown upon the treatment, and compel the discoverer to retreat into the background, and to seek irregular methods for making known his discovery. In the search of the blood for diseases of the body new methods have been evolved. By these methods we are shown that the natural secretions of the body yield signs of early and obscure disease, which the methods now in vogue fail to bring to light except when the disease is far advanced and often toy late to save life. The art of instantaneous microphotography is partially responsible for this new method as it demonstrates what the naked eye cannot see, and even shows such objects as the tubercular bacilli and the spores of syphilis floating in the serum of the blood. It would seem that every disease has its distinct representation in this organ. This can be discovered when the disease is in embryol and treated before the general system is impaired. How many physicians there are who are at their wits end to know from what their patients are suffering, because the disease is not sufficiently developed, who would gladly hail the knowledge which would lead them to a solution of the problem. The examination of the blood is nothing new, but the examining and photographing the fresh blood witnin a few seconds from the time it is drawn from the circulation is new. By this process the disease may be found before pathological change has been produced, and yet the discoverer of this method cannot have the advantage of a college laboratory because he has not a pull with the faculty. To get recognition in

America for discoveries like these it would seem necessary to first take them to Europe and have them returned with a foreign label.—*Dominion Dental Journal.*

